# Peripheral blood T-cell subset and its clinical significance in lupus nephritis patients

**DOI:** 10.1136/lupus-2022-000717

**Published:** 2022-08-16

**Authors:** Huijing Wang, Lan Lan, Jianghua Chen, Liang Xiao, Fei Han

**Affiliations:** 1 Kidney Disease Center, The First Affiliated Hospital, Zhejiang University School of Medicine, Hangzhou, Zhejiang, China; 2 Institute of Nephrology, Zhejiang University, Hangzhou, Zhejiang, China; 3 Key Laboratory of Kidney Disease Prevention and Control Technology, Hangzhou, Zhejiang, China; 4 Zhejiang Clinical Research Center of Kidney and Urinary System Disease, Hangzhou, Zhejiang, China

**Keywords:** lupus erythematosus, systemic, lupus nephritis, T-lymphocytes, helper-inducer

## Abstract

**Objectives:**

Lupus nephritis (LN) is a common and severe manifestation of SLE. Memory T (TM) cells have been implicated in the pathogenesis of SLE. This study aimed to investigate the clinical significance of T-cell subsets in a cohort of patients with LN.

**Method:**

The peripheral blood T cells of 24 patients with LN and 13 patients with idiopathic membranous nephropathy (iMN) were analysed by flow cytometry. SLE disease activity was evaluated by SLE Disease Activity Index-2000 (SLEDAI-2K). Patients with LN were followed up for >6 months.

**Results:**

Patients with LN presented lower frequency of CD4^+^ cells and higher percentage of CD8^+^ cells than patients with iMN, which was primarily due to lower CD4^+^ cell count. Interestingly, patients with LN under immunosuppressants had lower CD8^+^CD45RO^+^ TM frequency (p=0.007), fewer regulatory CD4^+^ T cells (p=0.04) than those without immunosuppressants. Most CD4^+^ and CD8^+^ TM cells in patients with LN showed an effector memory (CD45RO^+^CCR7^+^) phenotype. The frequency of CD8^+^CD45RO^+^ TM cells among the CD8^+^ T cells was negatively correlated with white blood cell count, haemoglobin, platelet and serum levels of complements C3 and C4, but was positively correlated with serum IgG, erythrocyte sedimentation rate and SLEDAI score (p<0.05 each). Consistently, the frequency of CD8^+^CD45RO^+^ TM cells was higher in patients with LN with positive antidouble-stranded DNA antibody, active renal disease, extrarenal manifestations and with sclerotic glomeruli or moderate-to-severe mesangial hypercellularity in renal pathology (p<0.05). Additionally, CD8^+^CD45RO^+^ TM cell frequency was significantly lower in patients with LN with renal complete remission than that in non-remission LN (18.7% vs 31.2%, p<0.05). None of these significant correlations was observed in CD4^+^ TM cells.

**Conclusion:**

The frequency of CD8^+^ TM cells correlates with disease activity and treatment response to immunosuppressant in patients with LN. CD8^+^ TM monitoring in patients with LN could provide more helpful indices for the monitoring and management of this disease.

**Key messages**

**What is already known about this subject?**

- SLE is associated with the memory T cell formation; however, the changes of memory T cells and its clinical significance in lupus nephritis (LN) remain unclear.

**What does this study add?**

- This study showed correlations of peripheral T cells and the frequency of CD8^+^CD45RO^+^ memory T cells with disease activity and response after immunosuppressive treatment in patients with LN.
- Patients with LN with high frequency of CD8^+^CD45RO^+^ memory T cells present high disease activity, more organs and systems, more severe renal injury and worse renal response.

**How might this impact on clinical practice?**

- These data suggest CD8^+^CD45RO^+^ memory T cells as a potential monitoring target in LN.
- Patients with LN with high frequency of CD8^+^CD45RO^+^ memory T cells may warrant more aggressive treatment and more close monitoring.

## Introduction

Lupus nephritis (LN) is the most common and severe organ-specific manifestation of SLE, characterised by the inflammatory infiltrates in kidney.^1^ Although the precise pathogenesis of LN remains unclear, disturbances in T lymphocyte has been implicated in this disease.

Increasing evidence indicates an important role of disproportionate T-cell subsets in SLE and related organ damage.^2 3^ Memory T cells (TM) are a dynamic pool of antigen-experienced T cells that accumulate over a lifetime. These cells can be further characterised into central memory (TCM) and effector memory (TEM) T cells by distinct homing capacity and effector function. As a potential diagnostic marker for active LN, CD8^+^ TEM cells infiltrate the kidney and can be even detected in the urine.^4 5^ Elevated CD8^+^ TEM frequency in patients with juvenile-onset SLE were persistently associated with active disease and a higher LN prevalence.^6^ Therefore, understanding the skewed phenotype and distribution of T lymphocytes and the memory subsets may potentially benefit clinical management of SLE and LN.

This study was aimed to determine the relationship between TM cells and the clinical status of patients with LN, and to evaluate the significance of TM cell quantity for treatment and renal remission.

## Materials and methods

This study was approved by the Ethics Committee of the First Affiliated Hospital of Zhejiang University School of Medicine. An extended materials and methods are available in the online supplemental section and supplemental table S1.

10.1136/lupus-2022-000717.supp1 Supplementary data

### Patient characteristics

Thirty-seven patients admitted in our centre between May and September 2021 were recruited into this study, including 24 patients who have LN and 13 patients with idiopathic membranous nephropathy (iMN). SLE and iMN diagnosis fulfilled the American College of Rheumatology classification criteria and 2012 Kidney Disease: Improving Global Outcomes criteria, respectively.^7 8^ Active LN was defined by new-onset proteinuria >0.5 g/g or active urinary sediment.^4^ Disease activity was evaluated by SLE Disease Activity Index Index-2000 (SLEDAI-2K).^9^ Remission was defined as urine protein-to-creatinine ratio (uPCR) <0.5 g/g and normal serum creatinine (SCr). Patients with iMN were recruited based on matched sex, age, uPCR, and SCr.

## Results

### Patients characteristics

As shown in table 1, 24 patients with LN were included, of which 8 (33.3%) involved haematology, 3 (12.5%) involved serosa, 3 (12.5%) involved musculoskeletal system and 3 (12.5%) involved skin. Their median SLEDAI score was 9. Twelve (50.0%) patients had positive anti-double stranded DNA (anti-dsDNA) antibody and 21 (87.5%) patients had decreased serum C3 level. Their uPCR was 3.3±5.9 g/g. Twelve (50.0%) patients did not receive any immunosuppressants before the study. Among these patients with LN, 14 (58.33%) were renal biopsy-proven, of which 6 (42.9%) presented sclerotic glomeruli, 7 (50.0%) presented crescents and 8 (51.7%) presented moderate-to-severe mesangial hypercellularity. Their median interstitial infiltrate rate was 10% (5%, 15%).

**Table 1:** Clinical characteristics of the patients with lupus nephritis (LN) and idiopathic membranous nephropathy (iMN)

Clinical characteristic	Patients with LN (n=24)	Patients with iMN (n=13)	P value
Sex, male/female	10/14	5/8	1.00
Age (years)	37 (25.8, 47.5)	47 (30.0, 54.0)	0.09
Disease duration (years)	3.0 (0.2, 8.5)		
New-onset, n (%)	9 (37.5%)	6 (50.0%)	0.73
SLEDAI median (range)	9 (8, 12.3)		
SLEDAI score ≥8, n (%)	20 (83.3%)		
Laboratory criteria			
Creatinine (µmol/L)	95 (61.0, 229)	82 (56.5, 98.5)	0.20
eGFR (mL/min/1.73 m^2^)	70.5±44.3	86.62±23.82	0.23
uPCR (g/g)	3.3±5.9	5.0±3.6	0.49
Albumin (g/L)	31.7±7.4	25.9±8.5	0.01
Erythrocyte sedimentation rate (mm/hour)	17 (7, 44.0)	35.5 (17.5, 44.0)	0.08
WBC, 10^9^/L	7.3±4.6	7.4±4.6	0.93
Haemoglobin (g/L)	103±23	117±26	0.12
Platelet, 10^9^/L	175±86	254±88	0.03
IgG (mg/dL)	1098±591	523±270	0.04
Autoantibodies and complement			
ANA positive, n (%)	19 (79.2%)		
Anti-dsDNA positive, n (%)	12 (50.0%)		
Anti-β2GP1 antibodies, n (%)	4 (16.7%)		
ACL, n (%)	6 (25.0%)		
Anti-Smith antibody, n (%)	5 (20.8%)		
Anti-SSA antibody, n (%)	13 (54.2%)		
C3 concentration (g/L）	0.6±0.3	1.0±0.6	
C4 concentration (g/L)	0.1±0.1	0.5±0.4	
Baseline SELENA-SLEDAI organ involvement			
Serosal, n (%)	3 (12.5%)		
Haematological, n (%)	8 (33.3%)		
Musculoskeletal, n (%)	3 (12.5%)		
Dermal, n (%)	3 (12.5%)		
Proteinuria ≥0.5 g/24 hours, n (%)	16 (66.7%)		
LN ISN category			
Class III, n (%)	2 (14.3%)		
class IV, n (%)	7 (50.0%)		
Class V, n (%)	2 (14.3%)		
Class III/IV+V, n (%)	3 (21.4%)		
Treatment			
Prednisone dosage ≥40 mg/day at baseline	11 (45.8%)	0	
Immunosuppressive drugs, n (%)	12 (50.0%)	7 (58.3%)	1.00
MMF	3 (12.5%)	0	
Tacrolimus	0	1 (8.3%)	
MMF+tacrolimus	1 (4.2%)	0	
CTX	0	2 (16.7%)	
RTX	1 (4.2%)	2 (16.7%)	
RTX+MMF/CTX/tacrolimus	3 (12.5%)	2 (16.7%)	
Belimumab	1 (4.2%)	0	
Belimumab+MMF	2 (8.3%)	0	

ACL, anticardiolipin antibody; Anti-dsDNA, anti-double stranded DNA; C3/4, complement 3/4; CTX, cyclophosphamide; eGFR, estimated glomerular filtration rate; MMF, mycophenolate mofetil; RTX, rituximab; SELENA-SLEDAI, Safety of Estrogens in Lupus Erythematosus National Assessment-SLE Disease Activity Index; uPCR, urine protein-to-creatinine ratio; WBC, white blood cell.

### T-cell subsets distribution

With a gating strategy shown in online supplemental figure S1, the naïve and memory population in both CD4^+^ and CD8^+^ T cells were defined according to the presence of memory marker CD45RO. CD4^+^ regulatory T (Treg) cells were gated by the expression of FOXP3. TCM and TEM cells were identified in patients with LN after CCR7 staining was added to the study.

**Figure 1:**
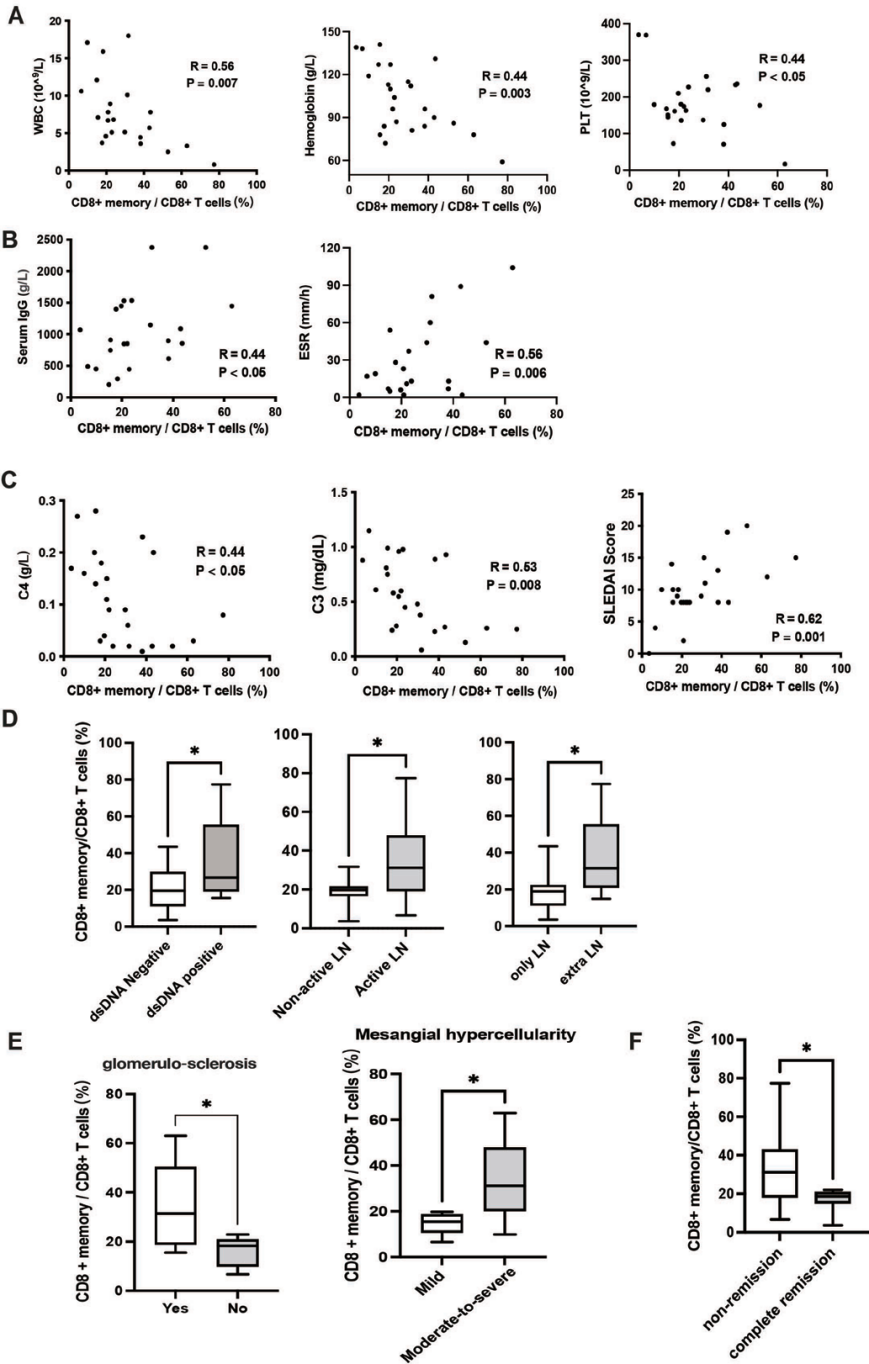
The correlations between the frequency of CD8^+^ memory T (TM) cells and clinical characteristics in patients with lupus nephritis (LN) (n=24). (A) Pearson’s correlations among CD8^+^ TM cells and the number of white blood cells (WBC), haemoglobin and platelets (PLT). (B) Pearson’s correlations among CD8^+^ TM cells and serum total IgG level, and erythrocyte sedimentation rate (ESR) level. (C) Pearson’s correlations among CD8^+^ TM cells and C3 and C4 level and SLE Disease Activity Index (SLEDAI) scores. (D) The frequency of CD8^+^ TM cells in LN with/without positive antidouble-stranded DNA (anti-dsDNA), in active/inactive LN, and in LN with/without extrarenal manifestations. (E) The frequency of CD8^+^ TM cells in LN with/without sclerotic glomeruli or moderate-to-severe mesangial hypercellularity. (F) The frequency of CD8^+^ TM cells in LN with complete remission (CR) (n=8) and LN without CR (n=11). *, P <0.05 with Student *t*-test (in panel D) or Mann-Whitney *U* test (in panels E and F).

As shown in online supplemental table S2, compared with patients with iMN, the number of CD4^+^ T cells in patients with LN tended to be lower (iMN 336.5/μL vs LN 164.2/μL, p=0.15), and CD8^+^ T cell levels are similar. These resulted in a significantly lower frequency of CD4^+^ T cells (iMN 62.4% vs LN 46.5%, p=0.02) and higher frequency of CD8^+^ T cells (iMN 29.0% vs LN 45.3%, p=0.02) in total T lymphocytes of patients with LN.

We further studied T cells from patients with LN according to the use of immunosuppressants. As shown in table 2, total CD4^+^ and CD8^+^ cell levels were similar in two groups. Interestingly, patients with immunosuppressants had lower percentages CD8^+^CD45RO^+^ TM (p=0.007) and CD8^+^ TEM (p=0.03), and a higher frequency of CD8^+^ naïve T cells (p=0.007). The frequency differences were likely to be caused by changes of both naïve and memory population. Such a naïve/memory ratio difference was not observed in CD4^+^ cells. However, patients with LN under immunosuppressants had fewer CD4^+^ Treg cells (p=0.04).

**Table 2:** The number of T-cell subsets in patients with LN with or without immunosuppressants

T-cell phenotype	Patients without immunosuppressants (n=12)	Patients with immunosuppressants (n=12)	P value
CD4^+^ (% lymphocytes)	48.5 (38.1, 71.1)	40.3 (22.8, 76.6)	0.39
CD4^+^ (cell/μL)	176.8 (30.0, 359.6)	161.3 (44.7, 471.7)	0.71
Naïve CD4^+^ (% CD4^+^)	58.3 (29.2, 85.1)	49.1 (24.8, 97.7)	0.81
Naïve CD4 (cell/μL)	75.7 (20.1, 171.3)	123.8 (19.2, 442.8)	0.58
CD4^+^ memory (% CD4^+^)	41.7 (14.9, 70.8)	50.9 (2.3, 75.2)	0.81
CD4^+^ memory (cell/μL)	74.0 (6.9, 231.8)	59.2 (8.0, 174.9)	0.62
CD4^+^ TCM (% CD4^+^)	10.5 (6.9, 16.9)	8.6 (7.0, 12.3)	0.46
CD4^+^ TCM (cell/μL)	21.2 (8.7, 29.2)	11.3 (3.8, 26.6)	0.19
CD4^+^ TEM (% CD4^+^)	53.1 (14.9, 120.6)	57.5 (16.4, 93.1)	0.88
CD4^+^ TEM (cell/μL)	78.5 (18.6, 151.0)	72.0 (20.5, 116.6)	0.77
CD4^+^ Treg (cell/μL)	1.8 (0.5, 3.6)	0.2 (0.2, 1.1)	**0.04**
CD8^+^ (% lymphocytes)	46.5 (18.6, 55.7)	42.4 (17.0, 66.3)	0.67
CD8^+^ (cell/μL)	133.2 (15.5, 352.5)	133.1 (50.7, 613.5)	0.58
Naïve CD8^+^ (% CD8^+^)	68.6 (40.1, 83.8)	81.8 (64.8, 95.8)	**0.007**
Naïve CD8^+^ (cell/μL)	86.6 (7.5,189.5)	99.7 (40.4, 559.5)	0.33
CD8^+^ memory (% CD8^+^)	31.5 (16.2, 59.9)	18.3 (4.2, 35.2)	**0.007**
CD8^+^ memory (cell/μL)	33.7 (7.8, 173.8)	20.64 (4.4, 93.5)	0.46
CD8^+^ TCM (% CD8^+^)	1.91 (0.2, 4.7)	0.54 (0.3, 1.0)	0.06
CD8^+^ TCM (cell/μL)	21.2 (8.7, 29.2)	0.98 (0.4, 3.9)	0.24
CD8^+^ TEM (% CD8^+^)	31.0 (13.5, 39.7)	19.5 (14.2, 21.4)	**0.028**
CD8^+^ TEM (cell/μL)	52.5 (12.9, 124.5)	62.5 (8.6, 84.3)	0.88

Data are expressed as medians (10th–90th percentile). P values less than 0.05 are presented in bold font.

The frequency of CD8^+^ TM was significantly lower, while the frequency of CD8^+^-naïve T cells was higher in patients with LN with immunosuppressants compared with those in patients with LN without immunosuppressants.

LN, lupus nephritis; TCM, central memory T cells; TEM, effector memory T cells; TM, memory T; Treg, regulatory T.

### CD8^+^ memory T cells are associated with LN active disease, renal injury and prognosis in LN

To investigate the clinical significance of TM cell subsets in patients with LN, we analysed the correlation between subset frequencies and clinical characteristics. The frequency of CD8^+^CD45RO^+^ TM cells in patients with LN was negatively correlated with the number of white blood cells (p=0.007), haemoglobin (p=0.003), platelet (p=0.03), and serum complements C3 (p=0.008) and C4 (p=0.03), but it was positively correlated with serum levels of IgG (p=0.04) and ESR (p=0.006), and SLEDAI score (p=0.001) (figure 1A–C). Consistently, the frequency of CD8^+^CD45RO^+^ TM cells was increased in patients with LN who had positive anti-dsDNA antibody, active renal disease and extrarenal manifestations (p=0.02) (figure 1D). Altogether, the high frequency of CD8^+^CD45RO^+^ TM cells was associated with active disease in patients with LN. However, CD4^+^CD45RO^+^ TM or CD8^+^ TCM subsets had no distinct correlation with clinical findings, but the frequency of CD8^+^ TEM was positively correlated with SLEDAI scores (p=0.04) (online supplemental figure S2-S4).

In the 14 patients with LN with renal biopsy, further correlation analyses were performed on T-cell subsets and pathological features. Interestingly, the frequency of CD8^+^CD45RO^+^ TM cells was significantly higher in patients with LN with sclerotic glomeruli or moderate-to-severe mesangial hypercellularity than that in patients with LN without the above changes (p=0.02) (figure 1E). For treatment response, 8 (42.1%) of 19 patients achieved complete remission in 6-month follow-up. The frequency of CD8^+^CD45RO^+^ TM cells was significantly decreased in patients with LN with renal complete remission than that in patients with LN with non-remission (18.7% vs 31.2%, p=0.03) (figure 1F).

## Discussion

This study identified a novel relationship between peripheral blood T-cell subsets and the clinical significance in patients with LN. Different CD4^+^ and CD8^+^ T-cell frequencies were observed between patients with LN and iMN, primarily due to the difference in CD4^+^ T-cell count. However, changes in CD8^+^ TM cell subset were associated with high disease activity, more involved organs and systems, more severe renal injury, and worse renal response in patients with LN. Patients with LN under immunosuppressants had lower frequency of CD8^+^ TM cells and fewer CD4^+^ Treg cell count.

Our current results indicate that disproportionate T-cell subsets are associated with the pathogenesis of SLE and LN. Early studies have reported increases in CD4^+^ TM cells and decreases in naïve CD4^+^ T cells in SLE, and also showed augmented CD4^+^ TEM cells, as well as the terminally differentiated TEM cells re-expressing CD45RA (TEMRA), in SLE.^2 3 10^ Different from most previous studies that compared patients with SLE or LN with healthy control subjects, we studied two groups of patients with different types of immune-mediated nephropathy. We found a lower level of CD4^+^ T cells in patients with LN when compared with iMN controls. This may due to the lymphopoenia commonly seen in patients with SLE.^6^ Decreases in cytotoxic cell counts and function in the peripheral of patients with SLE were observed and linked with SLE disease activity by Chen and Tsokos.^11^ Conversely, Robinson *et al* found an increase in total CD8^+^ T cells in patients with juvenile-onset SLE compared with healthy controls.^6^ In current study, we did not find difference in CD8^+^ cell counts between patients with LN and iMN. It is possible that total CD8^+^ cell count varies at different types or stages of SLE, but are indistinct between the two different nephropathies. Therefore, total CD8^+^ T-cell count is insufficient to serve as a valuable index.

Interestingly, changes in CD8^+^ TM subtypes, but not their CD4^+^ counterparts, correlate with the disease activity and immunosuppressant treatment response. In accordance with previous findings from patients with SLE,^2 6 12 13^ we further illustrated that frequency of CD8^+^ TM cells was positively correlated with LN disease activity, renal and extrarenal damage and treatment response. Unlike naïve CD8^+^ T cells, CD8^+^ TEM cells are matured effector cells with proinflammatory phenotypes.^14^ This enables them to mount a more rapid and potent immune reaction to repeated exposure of the same antigen.^14^ Due to the formation of autoantigen and pathogenic autoantibodies in SLE, a pool of autoreactive TM cells was steadily generated and maintained, which poses a considerable therapeutic challenge.^11 13 15^ We also found higher frequencies of CD8^+^ TM cells and CD4^+^ Treg cell count in the patients with LN without immunosuppressant treatment than those received, suggesting the immunosuppressants may counteract or reverse the disproportionate T-cell subsets in LN. Our data also suggest the CD8^+^ TM subtypes as useful indices when designing trials of LN progression, and as potential risk predictors in clinical practice.

The major limitation of our study is the relatively small size of the cohort and the finding needs further validation with a larger population of patients with LN and other disease controls. Despite a similar eGFR to patients with LN, the matched patients with iMN in our study presented worse nephrotic phenotypes than patients with LN, such as lower serum albumin. The lack of cross-sectional and validating pathological data and long-term visit data is another limitation. Additionally, the TEMRA cells, as a part of TEM cells, were not specifically studied in our flow cytometry analysis.

In conclusion, our study provides a novel insight into the T-cell subset distribution in LN. CD8^+^ TM cell subset may serve as a potential biomarker of disease activity and renal response after immunosuppressive treatment in LN. Patients with LN with high frequency of CD8^+^ TM cells warrant more aggressive treatment and closer monitoring. Therapies that depleting CD8^+^ TM cells would especially benefit these patients with LN, and monitoring CD8^+^ TM in patients with LN could provide more helpful indices when designing trials of disease progression.
