# Supplementary material for: Peripheral blood T-cell subset and its clinical significance in lupus nephritis patients
Source: Lupus Sci Med. 2022 Aug 16;9(1):e000717. doi: 10.1136/lupus-2022-000717 (PMC9386235; doi:10.1136/lupus-2022-000717)
Supplement: Supplementary data [file lupus-2022-000717supp001.pdf]

# Supplemental Materials

# Peripheral Blood T cells subset and its clinical significance in lupus nephritis patients

Huijing Wang, Lan Lan, Jianghua Chen, Liang Xiao*, Fei Han*

Kidney Disease Center, The First Affiliated Hospital, Zhejiang University School of Medicine; Institute of Nephrology, Zhejiang University; Key Laboratory of Kidney Disease Prevention and Control Technology, Zhejiang Province；Zhejiang Clinical Research Center of Kidney and Urinary System Disease, Hangzhou, Zhejiang, China

Correspondence to:

Fei Han

Kidney Disease Center, the First Affiliated Hospital, Zhejiang University School of Medicine, 79 Qingchun Rd, Hangzhou, Zhejiang Province, 310003, P.R. China.

Email: hanf8876@zju.edu.cn

Liang Xiao

Kidney Disease Center, the First Affiliated Hospital, Zhejiang University School of Medicine, 79 Qingchun Rd, Hangzhou, Zhejiang Province, 310003, P.R. China.

Email: xiaoliang@zju.edu.cn

*Running title: $CD8^+$ Memory T cells in LN*

## Method

### Patient characteristics

Twenty-four SLE patients with LN and thirteen patients with idiopathic membranous nephropathy (iMN) admitted in our center between May 2021 and September 2021 were included. SLE and iMN diagnosis fulfilled the American College of Rheumatology (ACR) classification criteria [7] and 2012 Kidney Disease: Improving Global Outcomes (KDIGO) criteria [8]. Active LN was defined by new-onset proteinuria >0.5 g/g or active urinary sediment [4]. Demographic and clinical data were obtained from the electronic medical records. Laboratory examination included serum anti-nuclear antibody (ANA), anti-double stranded (ds) DNA, anti-Smith, anti-cardiolipin antibody (ACL), and anti-β 2 glycoprotein 1 (anti-β2GP1), complete blood counts, serum albumin, serum creatinine (SCr), erythrocyte sedimentation rate (ESR), complement 3/4 (C3/C4), and serum total immunoglobulin (Ig) G, urine protein to creatinine ratio (uPCR). Disease activity score was evaluated by SLE Disease Activity Index (SLEDAI) score [9]. Remission was defined as uPCR < 0.5 g/g and normal SCr. In addition, 13 age-, sex, uPCR and SCr-matched iMN patients were recruited.

### Flow cytometry analysis

After EDTA-blood samples from patients were collected, 100 μL of each blood sample were used for flow cytometry analysis. In brief, red blood cells were lysed with 2 ml lysing solution for 10 min. The remaining cells were washed twice in wash buffer and then stained with a cell viability dye in PBS for 15 min at room temperature. Cell surface staining were performed with in a cocktail of antibodies, including anti-human CD3, CD4, CD8, CD45RO, and CCR7, in instructed dilutions for 25 min at 4C°. The cells were then fixed and permeabilized with a kit and intracellular staining of FOXP3 was performed. After staining, the whole single-cell suspension of each sample was aspired and analyzed flow cytometry (BD FACS Canto II) till the FACS tubes were empty. All reagents are listed in Table S1. Representative examples and the gating strategy of T cells and different subsets are shown in Figure S1.

## Statistical analysis

All the analyses were conducted by GraphPad Prism 9.0 and SPSS 22.0. Normally or non-normal distributed variables were analyzed by Student *t*-test or Mann-Whitney *U* test. The Pearson's correlation test was used for the analysis of clinical data and CD8+ TM cells. P values < 0.05 were considered statistically significant.

**Supplementary Table 1. The list of all antibodies and reagents for flow cytometry.**

| Reagents | Vender | Catalog number | Clone | Conjugated Fluorochrome |
|---|---|---|---|---|
| anti-human CD3 | BD Biosciences | 555332 | UCHT1 | FITC |
| anti-human CD4 | BD Biosciences | 560158 | RPA-T4 | APC/H7 |
| anti-human CD8 | BD Biosciences | 555369 | RPA-T8 | APC |
| anti-human CD45RO | BD Biosciences | 560608 | UCHL1 | PE/Cy7 |
| anti-human CD197 (CCR7) | BioLegend | 353220 | G043H7 | PerCP/Cy5.5 |
| anti-human FOXP3 | BioLegend | 320108 | 206D | PE |
| BD Pharm Lyse™ lysing solution (10X) | BD Biosciences | 555899 | | |
| Zombie Violet™ Fixable Viability Kit | BioLegend | 423114 | | |
| FIX & PERM™ Cell Permeabilization Kit | Invitrogen | GAS004 | | |

**Supplementary Table 2. The numbers of T-cell subsets in patients with lupus nephritis**

| T-cell phenotype | iMN patients (n=13) | LN patients (n=24) | P value |
|---|---|---|---|
| $CD4^+$ (% T lymphocytes) | 62.4 (28.4, 78.8) | 46.5 (27.3, 68.9) | **0.02** |
| $CD4^+$ (cell/μL) | 336.5 (47.0, 567.6) | 164.2 (45.0, 355.1) | 0.15 |
| naïve $CD4^+$ (% $CD4^+$) | 67.0 (20.1, 84.3) | 48.9 (25.9, 90.4) | 0.70 |
| naïve CD4 (cell/μL) | 192.2 (12.0, 391.7) | 81.9 (15.5, 197.3) | 0.29 |
| $CD4^+$ memory (% $CD4^+$) | 33.0 (15.7, 79.9) | 51.1 (9.6, 74.1) | 0.70 |
| $CD4^+$ memory (cell/μL) | 110.4 (11.8, 222.7) | 64.0 (8.0, 179.1) | 0.09 |
| $CD4^+$ TCM (% $CD4^+$) | | 10.5 (6.9, 21.7) | |
| $CD4^+$ TCM (cell/μL) | | 17.7 (6.3, 61.5) | |
| $CD4^+$ TEM (% $CD4^+$) | | 52.0 (15.6, 128.2) | |
| $CD4^+$ TEM (cell/μL) | | 73.4 (19.6, 160.5) | |
| $CD8^+$ (% T lymphocytes) | 29.0 (11.6, 58.8) | 45.3 (19.0, 57.2) | **0.02** |
| $CD8^+$ (cell/μL) | 143.6 (26.0, 330.0) | 128.1 (33.8, 421.7) | 0.75 |
| naïve $CD8^+$ (% $CD8^+$) | 76.3 (42.7, 92.4) | 77.6 (42.1, 91.7) | 0.78 |
| naïve $CD8^+$ (cell/μL) | 92.6 (19.9, 198.4) | 88.5 (9.3,333.8) | 0.70 |
| $CD8^+$ memory (% $CD8^+$) | 23.7 (7.6, 57.3) | 22.4 (8.3, 57.9) | 0.78 |
| $CD8^+$ memory (cell/μL) | 26.8 (3.8, 187.8) | 32.5 (7.4, 126.0) | 0.53 |
| $CD8^+$TCM (% $CD8^+$) | | 1.2 (0.2, 14.1) | |
| $CD8^+$ TCM (cell/μL) | | 2.9 (0.3,18.1) | |
| $CD8^+$ TEM (% $CD8^+$) | | 25.2 (13.9, 51.0) | |
| $CD8^+$ TEM (cell/μL) | | 52.5 (10.8, 152.2) | |

Data are expressed as medians (10th–90th percentile).

Abbreviations: TCM, central memory T cells; TEM, effector memory T cells.

In LN patients, an increase of $CD8^+$ cells and a reduction of $CD4^+$ cells, especially memory phenotype, were shown in comparison with idiopathic membranous nephropathy (iMN) patients. Circulating TM cells in LN patients frequently showed an effector $CD4^+$ and $CD8^+$ memory phenotype.

**Figure S1**

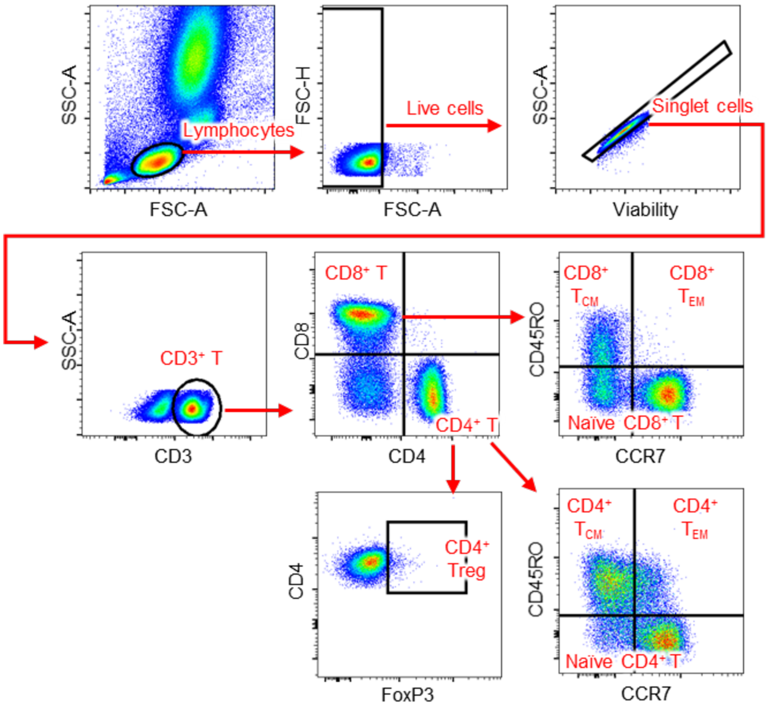


**Figure S1**. Flow cytometry gating strategy for T-cell subsets from a representative patient with lupus nephritis (LN). The naïve and memory population in both CD4$^{+}$ and CD8$^{+}$ T cells were defined according to the presence of memory marker CD45RO. CD4$^{+}$ regulatory T (Treg) cells were gated by the expression of FOXP3. TCM and TEM cells were identified in LN patients after CCR7 staining was added to the study.

**Figure S2**

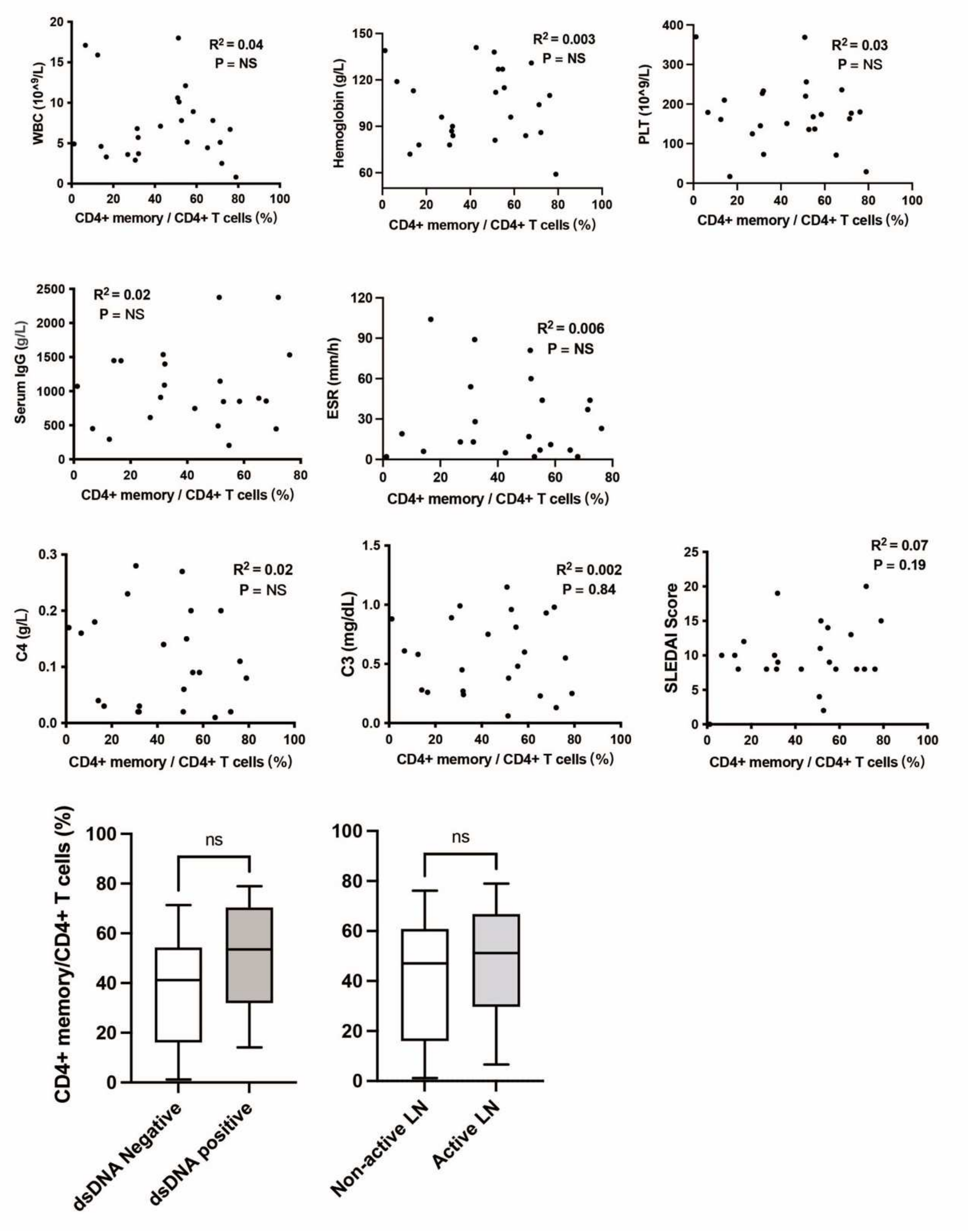


**Figure S2**. The relationship between the frequency of $CD4^+$ memory T (TM) cells and clinical characteristics in lupus nephritis (LN) patients (n = 24).

**Figure S3**

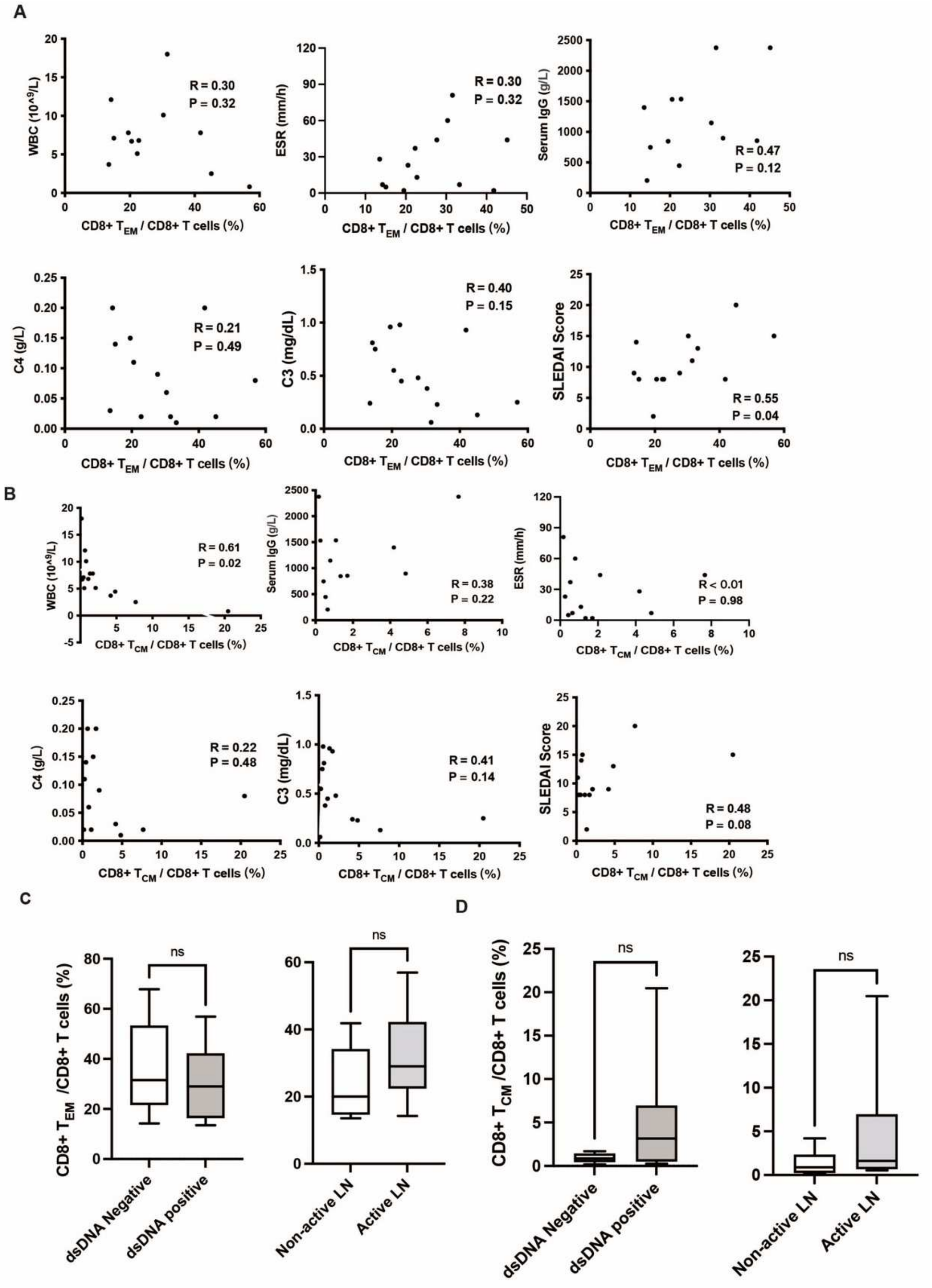


**Figure S3**. The relationship between the frequency of CD8⁺ central memory (TCM) / effector memory (TEM) T cells and clinical characteristics in lupus nephritis (LN) patients (n = 24). A, B. Pearson's correlations among $CD8^+$ TCM/TEM cells and the number of white blood cells (WBC), erythrocyte sedimentation rate (ESR) level, serum total IgG level, C3 and C4 level and systemic lupus erythematosus disease activity index (SLEDAI) scores. C, D. The frequency of $CD8^+$ TCM/TEM cells in LN with/without positive anti-dsDNA and in active/inactive LN.

**Figure S4**

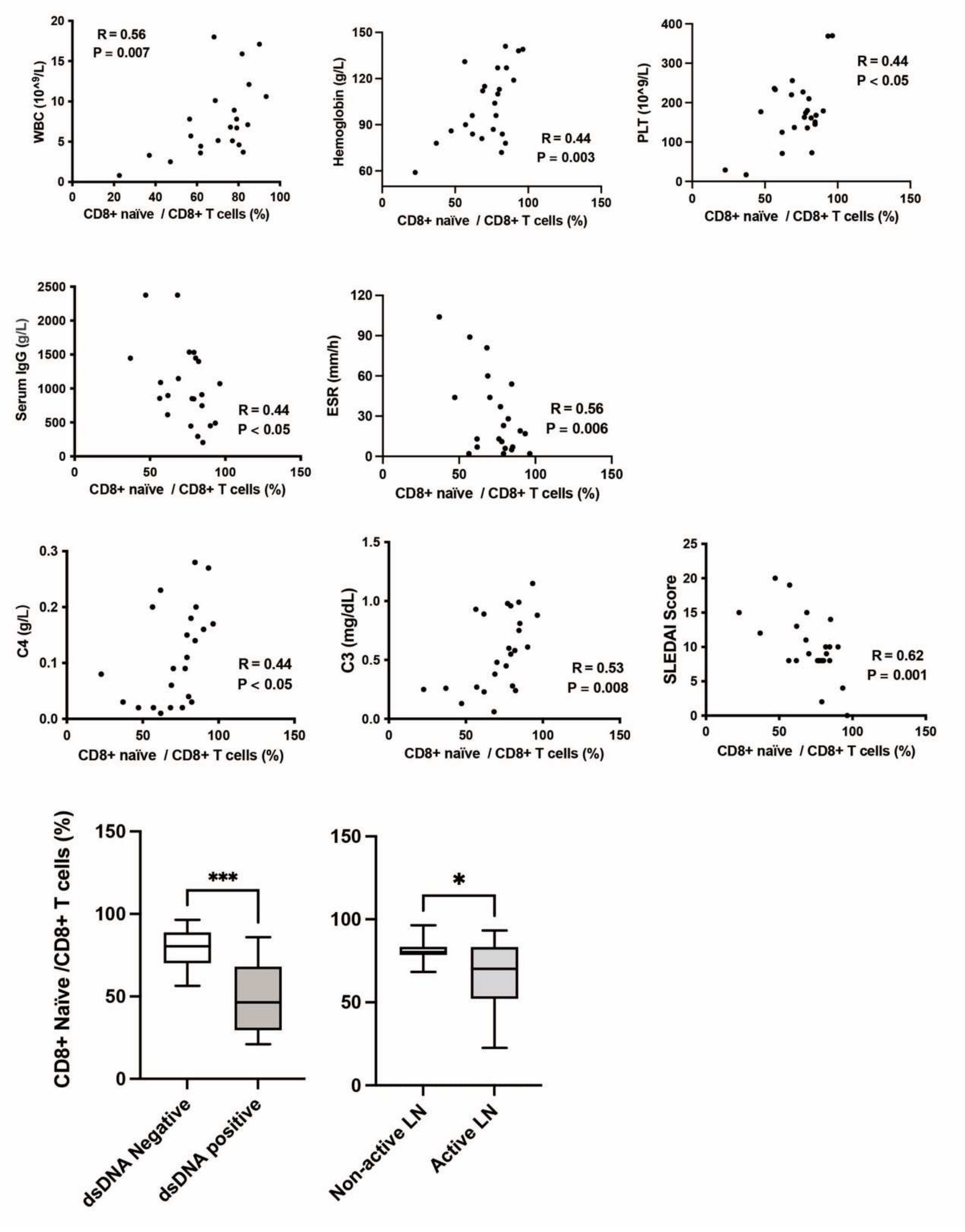


**Figure S4**. The relationship between the frequency of $CD8^{+}$ naïve T cells and clinical characteristics in lupus nephritis (LN) patients (n = 24).
